# The Static and dynamic functional connectivity characteristics of the left temporoparietal junction region in schizophrenia patients with auditory verbal hallucinations during low-frequency rTMS treatment

**DOI:** 10.3389/fpsyt.2023.1071769

**Published:** 2023-01-25

**Authors:** Yuanjun Xie, Muzhen Guan, Ying He, Zhongheng Wang, Zhujing Ma, Peng Fang, Huaning Wang

**Affiliations:** ^1^School of Education, Xinyang College, Xinyang, China; ^2^Department of Radiology, Xijing Hospital, Fourth Military Medical University, Xi’an, China; ^3^Department of Mental Health, Xi’an Medical University, Xi’an, China; ^4^Department of Psychiatry, Second Affiliated Hospital, Army Medical University, Chongqing, China; ^5^Department of Psychiatry, Xijing Hospital, Fourth Military Medical University, Xi’an, China; ^6^Department of Clinical Psychology, Fourth Military Medical University, Xi’an, China; ^7^Department of Military Medical Psychology, Fourth Military Medical University, Xi’an, China

**Keywords:** auditory verbal hallucinations, schizophrenia, fMRI, functional connectivity, rTMS, left temporoparietal junction region

## Abstract

**Background:**

Auditory verbal hallucinations (AVH) are a core symptom of schizophrenia. Low-frequency (e.g., 1 Hz) repetitive transcranial magnetic stimulation (rTMS) targeting language processing regions (e.g., left TPJ) has been evident as a potential treatment for AVH. However, the underlying neural mechanisms of the rTMS treatment effect remain unclear. The present study aimed to investigate the effects of 1 Hz rTMS on functional connectivity (FC) of the temporoparietal junction area (TPJ) seed with the whole brain in schizophrenia patients with AVH.

**Methods:**

Using a single-blind placebo-controlled randomized clinical trial, 55 patients with AVH were randomly divided into active treatment group (*n* = 30) or placebo group (*n* = 25). The active treatment group receive 15-day 1 Hz rTMS stimulation to the left TPJ, whereas the placebo group received sham rTMS stimulation to the same site. Resting-state fMRI scans and clinical measures were acquired for all patients before and after treatment. The seed-based (left TPJ) static and DFC was used to assess the connectivity characteristics during rTMS treatment in patients with AVH.

**Results:**

Overall, symptom improvement following 1 Hz rTMS treatment was found in the active treatment group, whereas no change occurred in the placebo group. Moreover, decreased static FC (SFC) of the left TPJ with the right temporal lobes, as well as increased SFC with the prefrontal cortex and subcortical structure were observed in active rTMS group. Increased dynamic FC (DFC) of the left TPJ with frontoparietal areas was also found in the active rTMS group. However, seed-based SFC and DFC were reduced to a great extent in the placebo group. In addition, these changed FC (SFC) strengths in the active rTMS group were associated with reduced severity of clinical outcomes (e.g., positive symptoms).

**Conclusion:**

The application of 1 Hz rTMS over the left TPJ may affect connectivity characteristics of the targeted region and contribute to clinical improvement, which shed light on the therapeutic effect of rTMS on schizophrenia with AVH.

## 1. Introduction

Auditory verbal hallucinations (AVH) are the most characteristic symptom of schizophrenia (1) and are defined as the conviction of hearing and perceiving “sound” without corresponding external stimulus input (2). Despite years of research, the neural mechanism of AVH continues to be unclear. Recently, neuroimaging studies have demonstrated the underlying functional and structural abnormalities associated with AVH in distributed brain regions, including Broca’s area, insula, hippocampal, and subcortical regions (3–6), which involved in speech processing, attention, and memory. The most consistent report is that the speech-processing regions of the superior temporal cortex are abnormal in terms of functional (7, 8) and anatomical (9, 10) alterations in schizophrenia with AVH.

Repetitive transcranial magnetic stimulation (rTMS) applies a repetitive pulsed magnetic field to the scalp, inducing an electric field in a discrete region of the brain (11), and thus alters the neuronal activity underneath the stimulate target (12). rTMS in low-frequency mode is thought to result in cortical inhibition (13). Low-frequency rTMS has been evident as a treatment for AVH. In the first study, Hoffman and colleagues indicated a reduction in AVH after 1-Hz rTMS targeting the superior temporal gyrus in schizophrenia patients compared to sham stimulation (14). Subsequently, numerous studies have replicated the preliminary finding (15–19). Meta-analysis studies have demonstrated a medium to a large effect size of low-frequency rTMS on AVH (20–22), although negative findings were reported (23, 24). The evidence supports the potential of low-frequency rTMS for the reduction of AVH. However, the underlying neural mechanisms of symptom improvement following rTMS treatment need further clarification.

Schizophrenia has been considered a dysconnectivity syndrome (25). And abnormal functional connectivity (FC) patterns between the distributed brain areas have been found in schizophrenia patients with AVH (26–28) and have been associated with the clinical severity of AVH (29). Interestingly, several studies have reported the effects of low-frequency rTMS on brain activity and connectivity in schizophrenia with AVH. In a small sample study, increased task-related activity in brain areas that involved in speech processing was observed in patients with AVH after 1 Hz rTMS treatment (30). Low-frequency rTMS mode also reduced brain metabolism in the left superior temporary gyrus and interconnected region, as well as enhanced metabolism in the contralateral cortex and the frontal lobes in schizophrenia patients with AVH (31).

Using the left temporoparietal junction area (TPJ) as seed, Vercammen and colleagues have found an increased FC between the seed and right insula in patients receiving 1 Hz rTMS applied to the left TPJ, while the sham stimulation group showed a decreased FC between the seed and left anterior cingulate (32). Recently, our study has demonstrated that low-frequency rTMS treatment in schizophrenia patients with AVH can induce the global and local topological properties changes of the whole brain functional network and were associated with the reduction of AVH (33). The results suggest that the application of low-frequency rTMS targeting the left TPJ may affect neural activity and connectivity in the targeted site and associated remote regions.

The purpose of present study was to investigate the underlying mechanism of 1 Hz rTMS treatment on schizophrenia with AVH using seed-based FC analysis. Using the left TPJ as seed, on the one hand, we replicated the results from the previous study (32) by using the static functional connectivity (SFC) analysis; on the other hand, provided Supplementary information by using seed-based dynamic functional connective (DFC) approach. We speculated that 1 Hz rTMS would induce beneficial static or dynamic FC (DFC) changes of left TPJ seed in patients with AVH after treatment and could be related to the improvement of clinical symptoms.

## 2. Material and methods

### 2.1. Participants

Fifty-eight patients were recruited in the study. Randomization of patients was performed outside the study group by simple random number generated from computer. Patients were allocated to one of the treatment conditions: rTMS stimulation to the left TPJ region (*n* = 32) or sham stimulation to the same site (*n* = 26). Supplementary Figure 1 shows the CONSORT flowchart with study enrollment, visits, and attrition. The diagnosis was confirmed by experienced psychiatrists according to the criteria of the Structured Clinical Interview for Diagnosis and Statistical Manual of Mental Disorder (DSM-V). The inclusion criteria for the patients met the follows: (1) AVH daily occurred with no less than two antipsychotic medications, and (2) at least five episodes of AVH per day during the past month. All patients received a stable dose of antipsychotic medications throughout the study period. All groups were matched on age, gender, education, and duration of illness. Exclusion criteria for all patients included the follows: (1) previous or current neurological disease, (2) history of head injury, (3) alcohol or drug abuse, and (4) contraindications to MRI scans.

Informed consent was obtained from all participants. The investigation was carried out by the Declaration of Helsinki and approved by the Medical Ethnic Committee of Xijing Hospital. This study was registered in China Clinical Trials (registration number: ChiCTR2100041876).^1^

### 2.2. Clinical measurements

The psychotic symptoms were evaluated by the positive and negative symptom scale (PANSS) (34), and the severity of AVH was assessed by the auditory hallucination rating scale (AHRS) (15). All clinical measures were executed by experienced psychiatrists at pretreatment and posttreatment for all patients.

### 2.3. rTMS procedure

Unlike our previous studies using case-control paradigm (33, 35–37), this study had a single-blind parallel design, only the rTMS administrator rather than researchers or raters knew the conditions to which patients were assigned. Stimulation was performed by a Magstim Rapid System (YIRUDE, Wuhan, China), using a 70 mm figure-of-eight coil. The stimulation site was determined based on the 10–20 international electrode location system (T3/P3). Active rTMS stimulation was carried out at 110% of the individual resting motor threshold (MT). The placebo condition was stimulated with a Magstim sham coil at the same location, which produced a similar sound stimulus, but no magnetic field through the skull. Each patient received a total of 15 rTMS sessions, each lasting 15 min, for a total of 9,000 pluses, over the course of 15 consecutive days.

### 2.4. Neuroimaging data acquisition and processing

Images were acquired on a 3.0-Tesla scanner (GE Medical Systems, Milwaukee, WI) with a standard 8-channel phased-array head coil. The images were scanned twice in patients (baseline and after treatment). Participants were instructed to stay awake and kept their eyes closed during the scanning duration. Functional images were collected using a gradient-echo-planar imaging sequence with the following parameters: repetitive time (RT) = 2,000 ms, echo time (ET) = 40 ms, matrix = 64 × 64, field of view (FOV) = 260 × 260 mm^2^, flip angle = 90°, 45 slices, with 3.5 mm slice thickness and no gap. A total of 210 whole brain volumes were acquired. An anatomical image was also obtained using an MP-RAGE sequence with the following parameters: TR = 8.1 ms, TE = 3.2 ms, matrix size = 256 × 256, flip angle = 12°, FOV = 240 × 240 mm^2^, 176 slices, and with 1.0 mm thickness (no gap).

Data were processed in Matlab 2018b platform (MathWorks, Natick, MA) using the DPABI toolbox.^2^ For each subject, the first 10 functional images were discarded and were then corrected for differences in slice timing and head motion. The corrected images were coregistered to the T1-weighted anatomical image. The coregistered anatomical images were segmented into gray matter, white matter, and cerebrospinal fluid. The following images were normalized into standardized Montreal Neurological Institute (MNI) space by the DARTEL algorithm and smoothed with a 6 mm Gaussian kernel. Subsequently, linear detrending and temporal filtering (0.01–0.1 Hz) were executed. Finally, nuisance covariates, including head motion, white matter signal, cerebrospinal fluid signal, and global mean signal, were regressed out.

### 2.5. Static functional connectivity analysis

**FIGURE 1:**
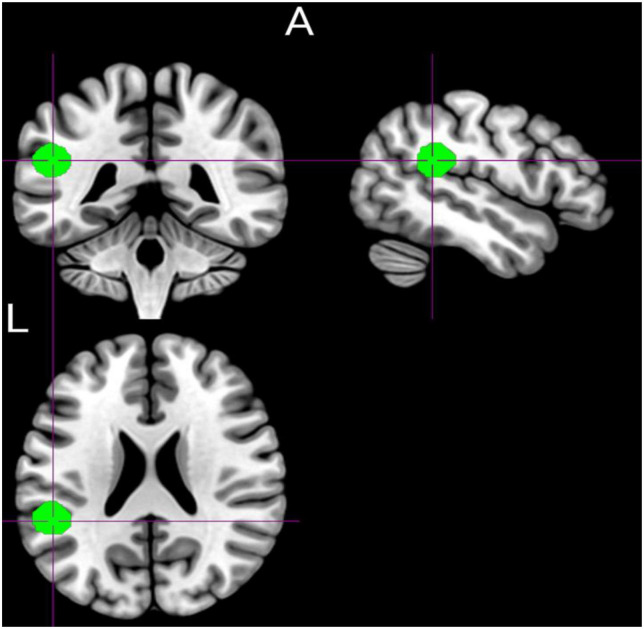
Graphical representation of the prior defined region of interest (left temporoparietal junction area) was defined using the Marsbar toolbox (MNI: −50.7, −41.4, 22.7).

Based on the previous study (32), the left TPJ seed was defined as a 10 mm radius sphere surrounding a central voxel (Figure 1). Resting-State fMRI Data Analysis (REST) toolkit (38) was used to calculate the seed-based resting-state SFC. The Pearson correlation coefficients between the time courses of the seed and the time series of each voxel of the whole brain were calculated. Correlation coefficients were transformed into Z-map with Fisher’s transformations to improve the normality of the data.

### 2.6. Dynamic functional connectivity analysis

The DFC was computed by using a sliding window approach through the DynamicBC toolbox (39). DFC calculates the correlation of time series according to a certain length of the sliding window. Based on the previous literature (40, 41), the length of the sliding window was set a 50 TRs, and the window overlap was set at 90%, resulting 31 windows for each participant. The temporal correlation coefficient between the time course of seed and time series all brain voxels within each sliding window was calculated and represented the DFC changes occurring entire scan course. Unlike the classic DFC studies that applied the k-means clustering algorithm to measure the frequency and structure of reoccurring FC patterns based on windowed covariance matrices between the predefined regions of interest (ROIs) or independent components (ICs) (42, 43), the variance of each voxel across all windows on seed-based DFC denoted the temporal variability in the strength of connectivity. Higher DFC variability indicated greater fluctuations of FC strength over time. Referring to the previous study (40, 44), the *Z*-valued FC variance maps were used in statistical analyses. To verity the results of DFC, the window size was changed with 40 TRs (overlap 90%) reanalyzed DFC for all participants.

### 2.7. Statistical analysis

Independent-sample *t*-test and chi-square test were used to test the clinical characteristic differences between the active treatment group and placebo group according to the nature of the data. In addition, a paired-sample *t*-test was used to test the changes in symptoms and FC between the patients before and after treatment. All results were controlled with age, sex, and head motion as covariates and were reported applied the Gaussian random field (GRF) correction with a voxel-level threshold of *p* < 0.05 and a cluster-level threshold of *p* < 0.05, the minus cluster size was set 30.

### 2.8. Correlation analysis

To investigate the associations between altered FC values and clinical responses, the mean values of altered SFC and DFC were extracted and then correlated with the scores of clinical responses with Pearson correlation analysis. A two-tailed p-level of 0.05 was considered as the criterion of statistical significance and corrected for multiple comparisons with the false discovery rate correction (FDR) method.

## 3. Results

### 3.1. Patient characteristics

Due to excessive head movement (head motion parameters are greater than 3.0 mm and 3.0 degrees), data from three patients had to be excluded, leaving 30 patients in the active treatment group and 25 patients in the placebo group. There were no statistical significances between the two group in demographical variables, including age (*t* = 0.644, *p* = 0.478), sex (χ^2^ = 0.045, *p* = 0.832), and education (*t* = 0.458, *p* = 0.756), and clinical measures, including illness duration (*t* = 0.525, *p* = 0.602), medication dosage (*t* = 0.218, *p* = 0.828), positive symptom (*t* = 0.983, *p* = 0.330), negative symptom (*t* = 0.178, *p* = 0.860), general symptom (*t* = 0.537, *p* = 0.593), and AVH (*t* = 0.689, *p* = 0.494). Details are displayed in Table 1.

**TABLE 1:** Demographic and clinical characteristics of the two patient groups receiving either rTMS or placebo treatment.

	Active group (*n* = 30)	Placebo group (*n* = 25)	*t*(χ^2^)	*p*
Age	30.30 ± 4.46	31.46 ± 6.35	0.644	0.478
Sex	17 (13)	14 (12)	0.045	0.832
Education (years)	13.20 ± 2.67	12.81 ± 2.71	0.458	0.756
Duration of illness (month)	21.36 ± 4.89	20.35 ± 3.38	0.525	0.602
Dosage (CPED, mg/day)	584.8 ± 152.39	573.46 ± 136.88	0.218	0.828
**PANSS**				
Positive symptom	19.65 ± 4.60	18.65 ± 3.36	0.983	0.330
Negative symptom	19.85 ± 4.53	19.35 ± 3.02	0.178	0.860
General symptom	40.35 ± 6.65	39.50 ± 4.62	0.537	0.593
AHRS	27.45 ± 6.14	25.73 ± 5.08	0.689	0.494

PANSS, positive and negative symptoms; AHRS, auditory hallucination rating scale; CPED, Chlorpromazine equivalent doses (45).

### 3.2. Treatment effect of rTMS

**FIGURE 2:**
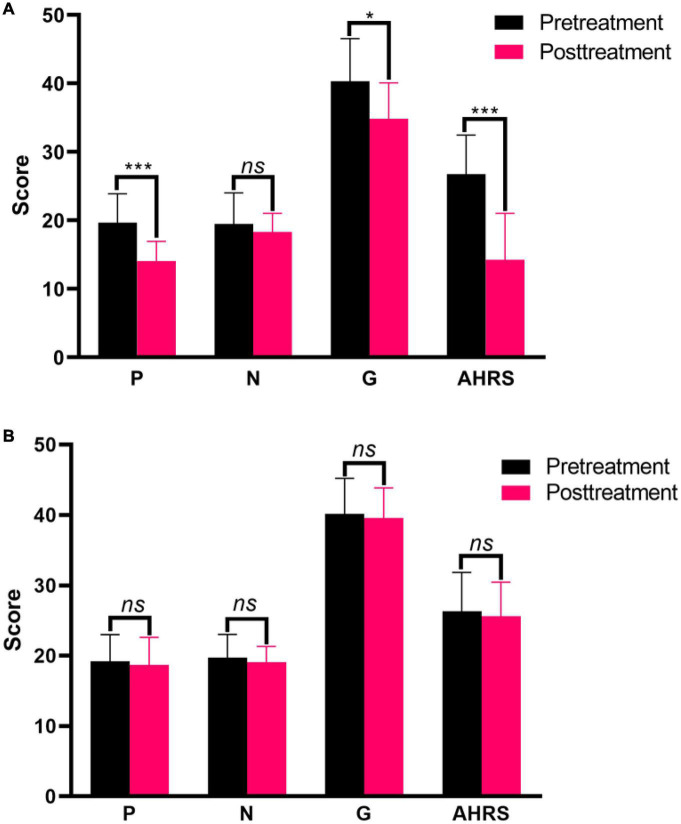
Clinical symptom responses between before and after treatment in the active treatment group **(A)** and placebo group **(B)**. *p < 0.05, ***p < 0.001. ns, non-significance.

Changes in clinical symptoms over time were assessed separately for two groups. Pretreatment and posttreatment measures showed significant decreases in positive symptom (*t* = 6.197, *p* = 0.000), general symptom (*t* = 2.661, *p* = 0.011), and AVH (*t* = 6.542, *p* = 0.000) scores in the active treatment group (Figure 2A). But the clinical measures showed no change at all in the placebo group over time (all *p* > 0.05) (Figure 2B).

### 3.3. Static functional connectivity analysis of left TPJ seed

**FIGURE 3:**
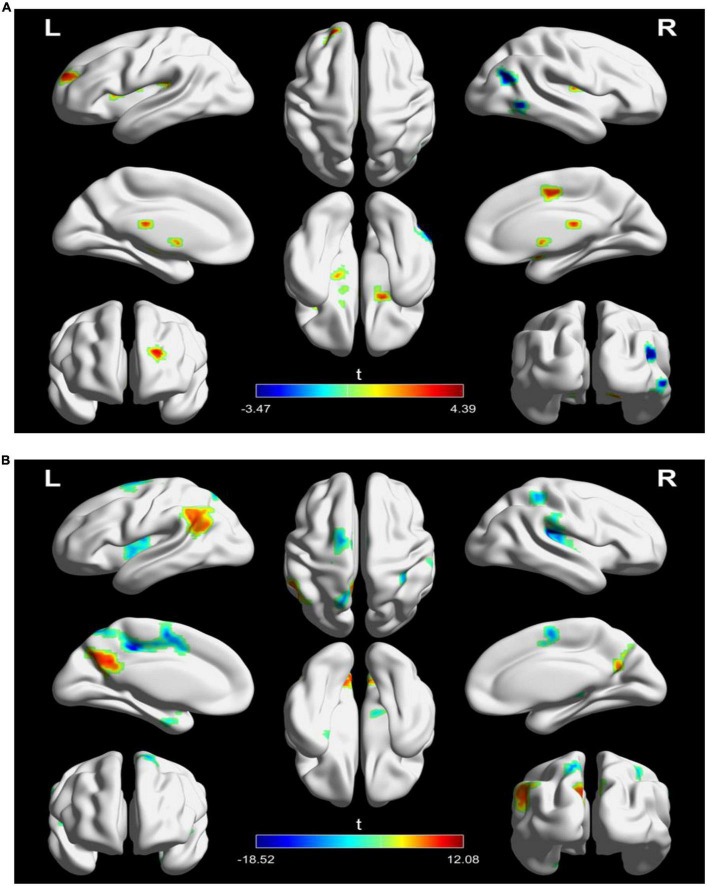
Significantly changes in static functional connectivity (SFC) of the left TPJ seed between before and after treatment in the two groups. **(A)** Significantly changes in SFC of the left TPJ seed in the active treatment group (posttreatment vs. pretreatment). **(B)** Significantly changes in SFC of the left TPJ seed in the placebo group (posttreatment vs. pretreatment). The warm color represents higher connectivity, and the cool color represents lower connectivity (GRF correction, *p* < 0.05, size > 30). TPJ, left temporoparietal junction; GRF, Gaussian random field.

For SFC analysis, the active treatment group showed significant FC changes in the left TPJ seed over the course of the treatment (Table 2 and Figure 3). Specifically, the patients in the active treatment group had increased FC with the right superior frontal gyrus, right supplementary motor, and bilateral putamen, as well as decreased FC with the right inferior temporal gyrus and right middle temporal gyrus (Figure 3A).

**TABLE 2:** Seed-based static functional connectivity group comparisons.

Group	Connectivity peak	Hemisphere	Size	MNI coordination			*t*-value
				**x**	**y**	**z**	
Treatment group (After vs. before)	Inferior temporal gyrus	R	64	60	−60	−15	-3.471
	Middle temporal gyrus	R	57	45	−66	24	-3.388
	Superior frontal gyrus	R	56	−24	57	27	3.689
	Supplementary motor area	R	45	12	−9	54	3.423
	Putamen	L	44	−18	15	−6	3.349
	Putamen	R	83	15	3	−6	4.392
Placebo group (After vs. before)	Angular	L	106	−57	−63	33	12.077
	Precuneus	L	175	−3	−78	42	11.151
	Middle cingulate gyrus	L	63	−12	−33	42	-16.230
	Insula	L	63	−39	−9	12	-12.413
	Insula	R	106	45	−30	18	-18.522

The placebo group on the other hand showed increased FC of the left TPJ with the left parietal cortex (e.g., angular and precuneus), as well as decreased FC of the left TPJ with left middle cingulate gyrus and bilateral insula (Figure 3A).

### 3.4. Dynamics functional connectivity analysis of left TPJ seed

**FIGURE 4:**
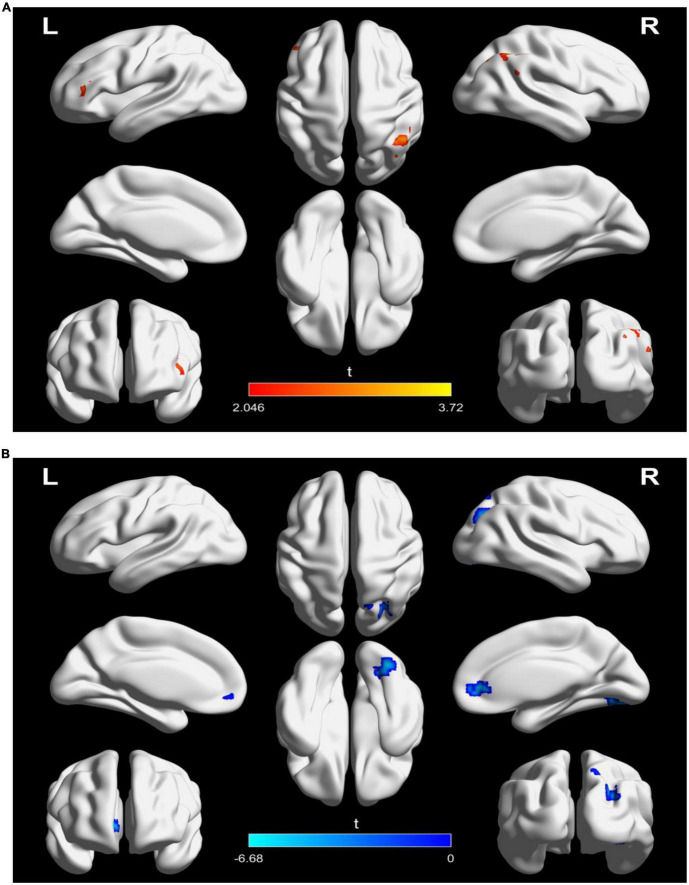
Significantly changes in dynamic functional connectivity (DFC) of the left TPJ seed between before and after treatment in the two groups. **(A)** Significantly changes in DFC of the left TPJ seed in the active treatment group (posttreatment vs. pretreatment). **(B)** Significantly changes in DFC of the left TPJ seed in the placebo group (posttreatment vs. pretreatment). The warm color represents higher connectivity, and the cool color represents lower connectivity (GRF correction, *p* < 0.05, size > 30). TPJ, left temporoparietal junction; GRF, Gaussian random field.

For DFC analysis, there was a significantly increased DFC between the seed and left inferior frontal gyrus and right inferior parietal lobule in the active treatment group after treatment (Table 3 and Figure 4A). But decreased DFC of the seed with the right inferior occipital gyrus, right anterior cingulate gyrus, and right superior parietal lobule was observed in the placebo group (Table 3 and Figure 4B).

**TABLE 3:** Seed-based dynamic functional connectivity group comparison.

Group	Connectivity peak	Hemisphere	Size	MNI coordination			*t*-value
				**x**	**y**	**z**	
Treatment group (After vs. before)	Inferior frontal gyrus	L	61	−48	45	3	3.321
	Inferior parietal lobule	R	117	45	−54	51	3.711
Placebo group (After vs. before)	Inferior occipital gyrus	R	48	30	−81	−12	−6.675
	Anterior cingulate gyrus	R	49	3	39	3	−6.367
	Superior parietal lobule	R	61	30	−75	51	−6.629

We performed a reproducibility analysis to verify that current findings reflected actual DFC changes in patients instead of difference of experimental parameters. For windows of 40 TRs with 90% overlap, we identified consistent alterations of DFC among the active treatment group and placebo group. The details are displayed in Supplementary Figure 2.

### 3.5. Correlation analysis results

**FIGURE 5:**
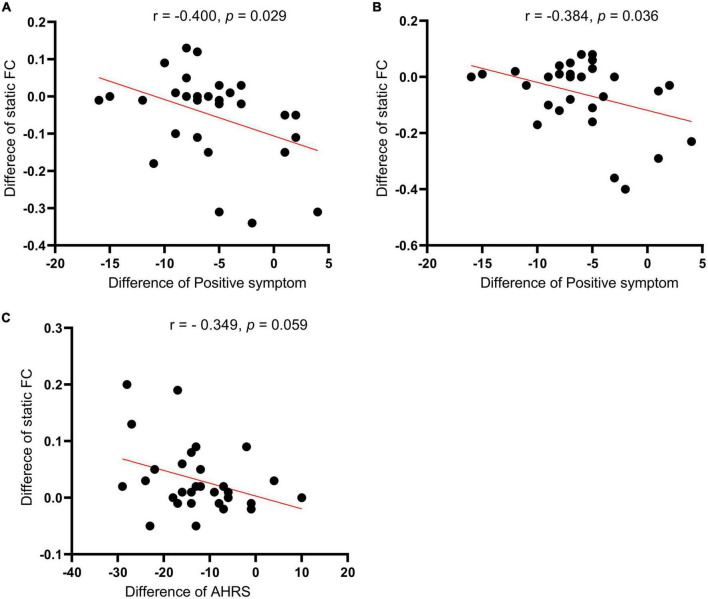
Correlations of the seed-based (left TPJ) static functional connectivity (SFC) difference values (posttreatment vs. pretreatment) in the left supplementary motor cortex **(A)** and right putamen **(B)** with the positive symptom score changes of PNASS in active treatment group. **(C)** Correlations of the seed-based SFC difference values (posttreatment vs. pretreatment) in the right inferior temporal gyrus with AVH score changes in active treatment group. TPJ, left temporoparietal junction; PANSS, positive and negative symptom scale; AHRS, auditory hallucination rating scale.

The correlation analysis showed that the SFC different values between posttreatment and pretreatment in the right supplementary motor cortex (*r* = −0.4000, *p* = 0.029) and right putamen (*r* = −0.384, *p* = 0.036) were significantly negatively with the positive symptom score changes of PANSS in the active treatment group, respectively (Figure 5). In addition, the SFC different value between after and before treatment in the right inferior temporal gyrus was marginally significantly correlated with the AVH score change in the active treatment group (*r* = −0.349, *p* = 0.059) (Figure 5). However, the correlations between the other static or DFC different values and clinical responses did not reach the statistically significant level (all *p* > 0.05).

## 4. Discussion

This study investigated the effects of l Hz rTMS on static and DFC in schizophrenia patients with AVH using the stimulation target (left TPJ) as seed. The results showed that active rTMS treatment could reduce the severity of clinical symptoms, whereas the placebo group appeared no symptom changes over the course of treatment. The results regarding alterations in clinical symptoms were fully consistent with the previous report (32). The results also agree with the meta-analysis studies (19, 20, 46) and confirmed the effectiveness of low-frequency rTMS treatment on AVH in schizophrenia with our previous studies (33, 35–37). Moreover, rTMS treatment induced beneficial connectivity changes of the seed in the active treatment group, including increased static FC (SFC) with prefrontal regions (e.g., right superior frontal gyrus and right supplementary motor cortex) and decreased SFC with the temporal lobe (e.g., right inferior temporal gyrus and middle temporal gyrus). In addition, increased DFC in the prefrontal (e.g., inferior frontal gyrus) and parietal (e.g., inferior parietal lobule) cortices was also observed in the active treatment group. But the placebo group showed a large proportion of reduced SFC or DFC of the seed after sham stimulation. In addition, these changed FC properties in the active treatment group were associated with a reduction in the severity of clinical symptoms (e.g., positive symptoms). These results were partially consistent with the previous study (32). Clinical characteristics and sample size of patients may be the main reasons for the differences in results. Our findings provided Supplementary information and suggested that induced broader FC changes relevant to the TPJ target site in schizophrenia patients could be the underlying neural mechanism of low-frequency rTMS treatment on AVH.

AVH may arise from a mismatch of inner speech and its attribution (47). The mismatch can be considered as a deficit in inner speech production or its perception and understanding (48). Functional imaging studies have explored the neural correlates of AVH and have suggested that experiencing AVH is primarily involved the language-related network associated with the Broca area, the anterior insula, the inferior frontal gyrus, the middle temporal gyrus, the superior temporal gyrus (4, 49). Although structural and functional neuroimaging evidence has suggested that schizophrenia is associated with reduced left-hemisphere language lateralization (50–52), the right hemisphere is apparently involved in the meditation of left hemisphere language function (53–55), since the disturbance in the interaction of hemispheres appears in schizophrenia (53). Previous studies have suggested that the inferior temporal gyrus and middle temporal gyrus are involved in language and semantic processing. Thus, the right inferior temporal gyrus and right middle temporal gyrus as left hemisphere homologs may play crucial roles in the pathophysiology of schizophrenia. AVH was associated with elevated metabolic activity and cortical involvement in the two regions (4, 56), which supports the hypothesis that AVH arises from the misinterpretation of inner speech and the aberrant activation of the langue-related areas.

There is evidence that schizophrenia is linked to dysfunction in distributed neural circuits (57, 58). rTMS has the potential to produce a distal effect that induces connection changes in remote brain regions beyond the stimulation site (59, 60) through long-term potentiation and long-term depression-like mechanisms (61). Studies have demonstrated that decreased metabolism in the auditor cortex, Broca area, and cingulate gyrus after TMS treatment relative to sham (62), which implies that the clinical effect of rTMS may come from a normalization of the hyperactivity of language regions involved in the emergence of AVH. In line with these findings, we found that low-frequency rTMS decreased the SFC of seed with the right temporal lobes (e.g., right inferior temporal gyrus and right middle temporal gyrus) in the active group and may suggest that the low-frequency TMS could have inhibitory effect on hyperconnectivity between the seed and language related areas, since rTMS in a low-frequency mode produce a reduction in cortical excitability of underlying brain tissue (63, 64). The previous studies suggest that rTMS might induce local changes at the stimulate site and adjacent remote areas (32, 65). Left TPJ as a critical stimulation target implicated in AVH generation and perception (3, 48). Then, decreased connectivity of the stimulate site with contralateral temporal lobes may decrease the likelihood of inner speech intrusions.

In contrast, increased SFC of the left TPJ seed with the brain areas, including the right superior frontal gyrus, right supplementary motor cortex, and bilateral putamen was observed in the active treatment group after rTMS stimulation. The low-frequency rTMS-induced strong connectivity with these areas might be explained by the release of transcallosal inhibition (66). For instance, enhanced activation is detected in the right hemisphere after left hemisphere injury (67). As we know, the superior frontal gyrus is located at the superior part of the prefrontal cortex and has been reported to be involved in a wide variety of cognitive and attention tasks (68, 69), which is a critical node of the cognitive control network (70). This cortex is associated with decreased cognitive control (71, 72) and decreased connectivity with other brain regions in schizophrenia patients (73, 74). The supplementary motor cortex participates in the preparation and execution of movements (75, 76), while the activation of this region is reduced during a motor task in schizophrenia patients (77) and may reflect the motor dysfunction in schizophrenia (78, 79), since motor symptom is one of clinical presentation of schizophrenia and frequently occur throughout the course of this disease (80, 81). In addition, the putamen is a subcortical structure that forms the basal ganglia and is involved in the regulation of perception and motor controls (82, 83). Degree centrality is decreased in the putamen in patients with AVH (84) and reflects abnormal connectivity of the putamen with the whole-brain network. Thus, the results mentioned above were agreed with the accumulating evidence (29, 85) and suggested that AVH are not solely associated with local brain dysfunction, but with abnormal neural networks, including cognitive control, motor, and perception processing systems.

However, low-frequency rTMS treatment over the left TPJ appeared to enhance the involvement of these dysfunctional brain regions and help restore their normal functions. Specifically, the putamen is rich in dopaminergic neurons, and abnormal dopaminergic transmission in the putamen is related to positive symptoms such as hallucinations (86). Therefore, increased connectivity with the putamen after active rTMS treatment in patients could normalize the dopaminergic activity of basal ganglia and contribute to the reduction of positive symptoms. Moreover, the supplementary motor cortex is involved in the imaging of speech in addition to movements (87). During an auditory verbal imagery task (imaging a sentence being spoken by another person), which requires generating and monitoring inner speech, schizophrenia patients with AVH had reduced activation in the supplementary motor area (88). The authors clarify that a predisposition to verbal hallucinations may be related to an inability to activate areas involved in monitoring inner language. In addition, the inferior temporal gyrus subserves language and sematic processing (89). Patients with AVH had increased local spontaneous neural activity in the inferior temporal gyrus (90). Thus, functional abnormalities in the cortices may cause a mismatch between perceived and predicted outcomes of intrinsic speech activity and associate with the experience of AVH. We found that rTMS treatment significantly (or marginally) induced the alternated connectivity of the TPJ with the right inferior temporal gyrus and supplementary motor cortex, and then could result in a restoration of the cortical functions and a reduction in the severity of clinical symptoms.

The human brain works in a dynamic approach to integrate, coordinate, and respond to external and internal stimuli across multiple time scales (43). Thus, DFC can capture the wealth of information contained within the time-varying features in interregional functional interactions and reveal additional alternations in schizophrenia that could not be discovered by SFC (91, 92). We found that increased connectivity of the seed with the left inferior frontal gyrus and right inferior parietal lobule were detected only by the DFC approach in the active treatment group after rTMS stimulation. The left inferior frontal gyrus is involved in langue processing. Underactivation of the left inferior frontal gyrus has been reported in patients with schizophrenia during various langue tasks, including verbal learning and speech comprehension (93), and semantic encoding (94). The inferior parietal lobule is known to be a central hub of multisensory integration (95). In schizophrenia, neuroimaging studies have found that the inferior parietal has reduced cortical thickness (96), functional activation (97), and hemispheric asymmetry (98). Deficits in this area can cause perception dysfunction in schizophrenia (99, 100). The enhanced synchronization of the seed with these two regions as detected by DFC in the active treatment group might be beneficial for the recovery of speech and perceptional functions. These findings provided a comprehensive understanding of the treatment effect of rTMS treatment in schizophrenia.

Despite wed did not observe any clinical improvements after placebo treatment, some alternations based on the SFC and DFC analyses were presented in the placebo group, similar to the previous study (32). The decreased connectivity of the seed with the most brain regions, including the cingulate cortex, insula, superior parietal lobule, and inferior occipital lobe, was observed, which may represent the continuous deterioration of clinical symptoms over time, since hypoconnectity is dominant in schizophrenia (101, 102) and AVH (103, 104), and these regions critically represent the pathophysiology of schizophrenia (105–107) as well as AVH (49, 108–110). On the contrast, the placebo group also had increased connectivity of the seed with certain parietal areas (e.g., left angular and left precuneus) by SFC analysis. Abnormal functional (e.g., hypoactivation) (111, 112) and structural (e.g., reduced gray matter volume) (113, 114) alternations in the left angular and left precuneus have been reported in schizophrenia and appeared to be correlated with the severity of AVH (115, 116). Thus, the increased synchronization in these brain regions underlies core deficits of schizophrenia and may imply a neural compensation to overcome the primary functional defects in patients with AVH. Nevertheless, the results suggested that future studies may need to adequately seek the placebo effects and real rTMS effects by directly comparing placebo-responders and rTMS responder following rTMS treatment.

## 5. Conclusion

In summary, we observed that clinical improvement in the active treatment group coincided with FC alternations of the TPJ seed over the course of rTMS treatment. Low-frequency rTMS treatment of the left TPJ area could modulate neural circuits implicated in AVH, which contribute to the clinical improvement in schizophrenia.

## Data availability statement

The original contributions presented in this study are included in the article/Supplementary material, further inquiries can be directed to the corresponding authors.

## Ethics statement

The studies involving human participants were reviewed and approved by the Medical Ethics Committee of the Xijing Hospital. The patients/participants provided their written informed consent to participate in this study.

## Author contributions

YX, MG, and ZW designed and organized the research. YX, YH, ZM, and ZW collected the imaging and clinical data. YX and PF analyzed the data. YX, MG, and YH wrote and revised the manuscript. PF and HW supervised the project and provided fund support. All authors contributed to the article and approved the submitted version.

## Footnotes

1
http://www.chictr.org.cn/

2
http://rfmri.org/dpabi

## Supplementary material

Supplementary Figure 1 The flowchart of study.

Supplementary Figure 2 Significantly changes in dynamic functional connectivity (DFC) (window of 40 TRs with 90% overlap) of the left TPJ seed between before and after treatment in the two groups. **(A)** Significantly changes in DFC of the left TPJ seed in the active treatment group (posttreatment vs. pretreatment). **(B)** Significantly changes in DFC of the left TPJ seed in the placebo group (posttreatment vs. pretreatment). The warm color represents higher connectivity, and the cool color represents lower connectivity (GRF correction, *p* < 0.05, size > 30). TPJ, left temporoparietal junction; GRF, Gaussian random field.
