# Supplementary figures and images for: The Static and dynamic functional connectivity characteristics of the left temporoparietal junction region in schizophrenia patients with auditory verbal hallucinations during low-frequency rTMS treatment
Source: Front Psychiatry. 2023 Jan 25;14:1071769. doi: 10.3389/fpsyt.2023.1071769 (PMC9907463; doi:10.3389/fpsyt.2023.1071769)

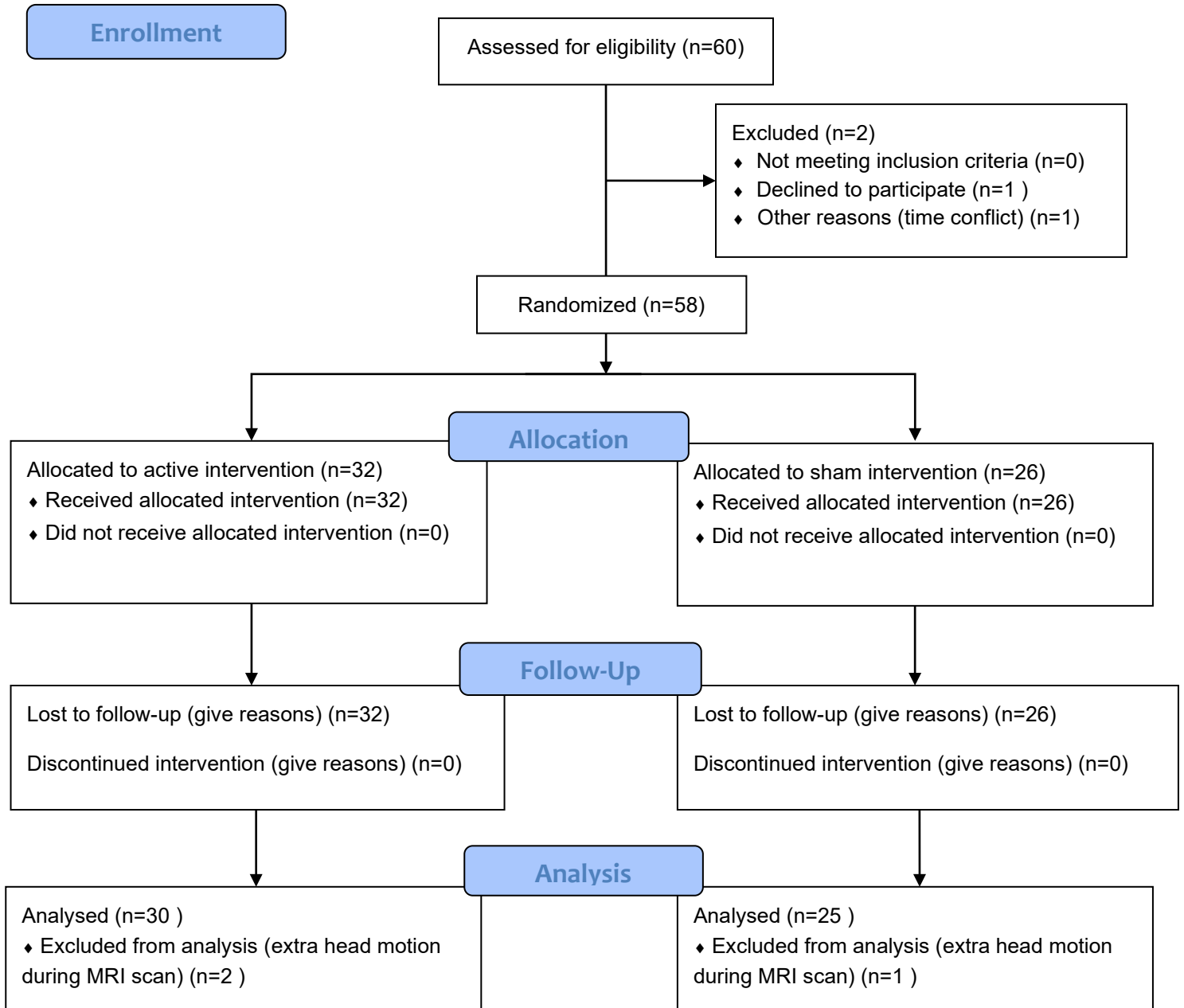

**Supplementary Figure 1** The flowchart of study

Supplement: Supplementary Figure 1 — The flowchart of study. [file Image_1.pdf]

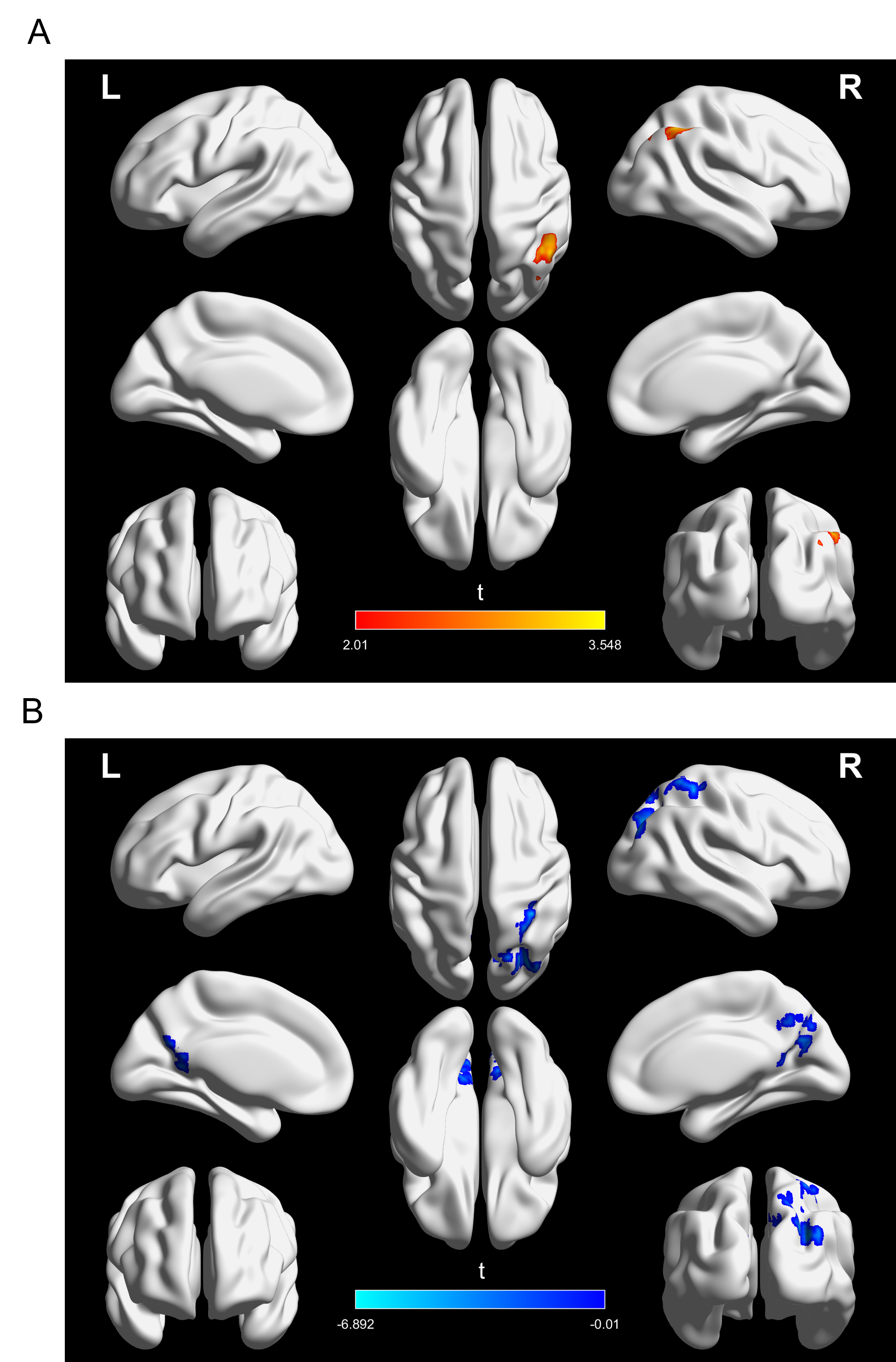

Supplement: Supplementary Figure 2 — Significantly changes in dynamic functional connectivity (DFC) (window of 40 TRs with 90% overlap) of the left TPJ seed between before and after treatment in the two groups. (A) Significantly changes in DFC of the left TPJ seed in the active treatment group (posttreatment vs. pretreatment). (B) Significantly changes in DFC of the left TPJ seed in the placebo group (posttreatment vs. pretreatment). The warm color represents higher connectivity, and the cool color represents lower connectivity (GRF correction, p < 0.05, size > 30). TPJ, left temporoparietal junction; GRF, Gaussian random field. [file Image_2.tif]
